# Evaluating the social fitness Programme for older people with cognitive problems and their caregivers: lessons learned from a failed trial

**DOI:** 10.1186/s12877-018-0927-8

**Published:** 2018-10-04

**Authors:** H W Donkers, D J Van der Veen, S Teerenstra, M J Vernooij-Dassen, M W G Nijhuis-vander Sanden, M J L Graff

**Affiliations:** 1 Radboud university medical center, Radboud Institute for Health Sciences, IQ healthcare, P.O. Box 9101, 6500 HB Nijmegen, The Netherlands; 2 Radboud university medical center, Donders Institute for Brain, Cognition and Behaviour, Radboudumc Alzheimer Center, Nijmegen, The Netherlands; 3 Department for Health Evidence, section Biostatistics, Radboud university medical center, Radboud Institute for Health Sciences, Nijmegen, The Netherlands; 4 Department of Rehabilitation, Radboud university medical center, Donders Institute for Brain, Cognition and Behaviour, Nijmegen, The Netherlands

**Keywords:** Cognitive functioning, Process evaluation, Psychosocial care, Social health, Social participation

## Abstract

**Background:**

This process evaluation article describes the lessons learned from a failed trial which aimed to assess effectiveness of the tailor-made, multidisciplinary Social Fitness Programme to improve social participation of community-dwelling older people with cognitive problems (clients) and their caregivers (couples).

**Methods:**

A process evaluation was performed to get insight in 1) the implementation of the intervention, 2) the context of intervention delivery from professionals’ point of view, and 3) the potential impact of intervention delivery from participants’ perspectives. Data was gathered using mixed-methods: questionnaires, focus group discussions, interviews, medical records.

**Results:**

*1) Implementation.* High study decline (65,3%) was mainly caused by a lack of internal motivation to increase social participation expressed by clients. 17 couples participated, however, intervention delivery was insufficient. *2) Context.* Barriers during intervention delivery were most often related to client (changing needs), caregiver (increased burden) and health professional factors (delivery of integrated care lacked routine). *3) Impact* Qualitative analyses revealed participants to be satisfied with intervention delivery, we were unable to capture these results through our primary outcome measure.

**Conclusions:**

This process evaluation revealed the Social Fitness study did not fit in three ways. First, framing the intervention on social participation promotion was as threatening to clients. The feeling of being unable to adequately contribute to social interactions seemed to be causing embarrassment. Second, the intervention seemed to be too complex to implement in the way it was designed. Third, there is a tension between the offering of a personalised tailor-made intervention and evaluation through a fixed study design.

**Trial registration:**

The trial which is evaluated in this article (the Social Fitness study) is registered with the Dutch Trial Register (NTR), clinical trial number NTR4347.

**Electronic supplementary material:**

The online version of this article (10.1186/s12877-018-0927-8) contains supplementary material, which is available to authorized users.

## Background

Social participation is a central theme in psychosocial dementia care [1], and as part of social health it is considered important for successful and healthy ageing [2, 3]. The definition for social participation used in this study is: involvement in social activities in which there is interaction with others in the society which makes one feels valued, attached to the community and gives meaning to someone’s life [4–6]. Six levels of involvement are: 1) doing an activity in preparation for connecting with others, 2) being with others, 3) interacting with others without doing a specific activity with them, 4) doing an activity with others, 5) helping others, and 6) contributing to society [4]. Social participation is a potentially modifiable factor, however, effective person-centred interventions focused on social participation promotion for people with cognitive problems and their caregivers are scarce [7, 8]. We therefore developed the Social Fitness Programme (SF Programme); an intervention aimed at enabling social participation [9].

The SF Programme is a tailor-made multidisciplinary intervention combining guidance by an occupational therapist (OT; applying the evidence-based COTiD-intervention [10, 11]) physiotherapist (PT; through the evidence-based person-centred Coach2Move-protocol [12–14]) and welfare professional [9]. The SF Programme combines active treatment methods, including exercises and training of bodily functions and the effective use of skills and strategies to improve participation in social activities of the client and caregiver. Our starting point was to incorporate effective elements of psychosocial interventions in dementia as the preconditions in the Social Fitness Programme. The *multi-component* [15, 16] intervention therefore is aimed at empowering and enabling clients and caregivers to participate socially through a *patient-centred* [17, 18] approach. This *community-based* [19] intervention consists of a *tailor-made* intervention plan which includes *feasible goals* [20, 21] that represent the social activities which are relevant and important to the individual person. To achieve this, *shared-decision making* principles are incorporated during goal setting and intervention delivery [22–24]. Intervention delivery takes place in the *own environment* to enable the removal of barriers and to facilitate the execution of activities in the social and physical environment (*the context*). The professionals use a personalised approach to *empower* participants to *optimise compensatory and environmental strategies* and make *use of adaptations* to enable clients and caregivers to participate socially in their own context. The intervention addresses *needs, preferences and abilities* of the person with cognitive problems, the caregiver and their social environment. The professionals involved in intervention delivery used coaching methods focused on improving their self-confidence and self-management. The welfare professionals provided practical support in achieving participants’ goals, such as active guidance towards clients’ activities and caregiver support.

Enhancing social participation of people with cognitive problems and their caregivers is challenging, as our previous studies [9, 25] revealed. We found barriers, on acceptability, demand, implementation and practicability. Also, interdisciplinary collaboration between healthcare and welfare professionals was suboptimal. However, the Social Fitness Programme seemed feasible according to stakeholders and limited efficacy showed promising results: 78,6% percent of the participants with cognitive problems attended new (social) activities during the SF Programme, with or without their caregivers. However, we found barriers influencing feasibility [26].

While results on feasibility were promising, we aimed to perform a Randomised Controlled Trial (RCT) as suggested in the Medical Research Council (MRC) guidance [27, 28]. We aimed to include 92 couples for a full RCT; however after an inclusion period of 15 months it appeared that 32 couples declined participation and only 17 were included. Recruitment difficulties are often seen in research. As a result, recruitment is often slower than expected and required sample sizes are not obtained within funding deadlines. This makes under-recruitment a problem or trials with negative consequences for patients, science and economy [29–33].

As a result of the high amount of study decline in relation to the participants who gave informed consent, study inclusion was terminated. Additional file 1 contains the study protocol of the trial and descriptive results of our primary and secondary outcomes. To get insight in the implementation of the intervention, the context of intervention delivery from professionals’ point of view, and the potential impact of intervention delivery from participants’ perspectives, we performed a mixed-method process evaluation according to the guide by Saunders and colleagues [34]. This article describes this process evaluation and the lessons learned from a failed trial.

## Methods

### Study design

We used a mixed-methods design for our process evaluation. Data for this process evaluation was gathered in parallel to the effectiveness study through questionnaires, focus group discussions, face-to-face and telephone interviews, and medical records. Data was gathered at different moments: before the start of the intervention, during intervention delivery, and after study termination.

We applied a comprehensive and systematic approach in which we focussed on three areas: implementation, context of intervention delivery and impact of intervention delivery [34]. *Implementation* captures the process of intervention delivery and consists of different elements, including: reach (participation rate), recruitment of participants (reasons to participate and reasons to decline participation), intervention adherence (dose delivered and dose received), fidelity (the quality of intervention delivery) and adaptations (changes that undermine intervention fidelity). The *context* of intervention delivery refers to external elements influencing implementation or effects, both positively and negatively. The potential *impact of intervention delivery* on participants was investigated from both clients and caregivers point of view.

### Participants

*People with cognitive problems* (clients) were eligible for study participation if they lived at home, wished to improve their social participation and suffered from cognitive problems defined as: dementia diagnosis (Mini-Mental State Examination; MMSE ≥10; [35] or memory problems signalled by the referring professional (MMSE 10–24) or with a primary caregivers’ score of ≥3.6 on the Informant Questionnaire on COgnitive Decline in the Elderly (IQCODE-N [36] (only for clients with high intelligence or high levels of education resulting in an MMSE-score between 25 and 30). Moreover, also *clients’ primary caregivers* who wished to maintain or improve their own social participation or the social participation of the people they cared for were eligible. Participants’ wish to improve their social participation was established during intake by their ability to formulate at least one social participation goal on level two (being with others) of our operational definition for social participation.

### Intervention and implementation

The SF Programme is a multidisciplinary intervention which consists of an integration of community occupational therapy (OT) following the Community Occupational Therapy in Dementia (COTiD) programme [10, 11, 37], physiotherapy (PT) following the Coach2Move programme [12–14], and guidance by welfare professionals. For a more detailed description see our feasibility study [9]. After a thorough problem analysis by OT and PT, the OT discussed with the client and caregiver personal goals, including goals on social activities. The OT consecutively discussed with the PT and welfare professional what was needed in the intervention and support to reach these goals, and converted this information into an intervention plan. The intervention plan included a combination of information and instruction combined with exercises to improve the use of strategies, skills, bodily functions and movement capacity using coaching methods focused on improving the self-confidence, self-efficacy, and self-management skills. Clients were supported and trained to use compensational strategies effectively, and caregivers were supported and trained in problem solving and communication skills. Welfare professionals aimed to elicit positive experiences in social activities, by guiding participants towards activities that were tailored to personal motivation, routines and abilities, and to enhance their personal and environmental resources. The SF Programme was goal-oriented and contained up to two interdisciplinary professional home visits a week during three months, and less frequent continued guidance after three months of intervention. During the multidisciplinary intervention, the General Practitioner (GP) and other professionals continued to provide primary care as usual.

### Study procedures and data collection

Table 1 provides an overview of the data collection. 1. Implementation: *Reach and recruitment* of participants were evaluated by research assistant through analysing records from telephone interviews with referring professionals and with people who seemed willing to participate. *Intervention adherence* was determined before the start of the intervention by using case vignettes, and after intervention delivery by assessing the medical records using a predefined checklist. The case vignettes included a case description based on a real case. OTs and PTs were asked to answer open ended questions regarding problem analysis, goal setting, and interdisciplinary cooperation. To evaluate *fidelity and adaptations* OT and PT medical records of participants allocated to the intervention group were studied. Involvement of welfare professionals was addressed as part of OT records.

**Table 1:** Mixed method process evaluation of the Social Fitness Programme

Focus and operationalisation	Method	Analysis
1.Implementation *Reach & Recruitment: Reasons for participation and reasons to decline participation.*	Records from telephone interviews	Content analysis of records from telephone interviews by research assistant with referring professionals and with people who seemed willing to participate.
*Adherence: Intervention dose delivered and intervention dose received by participants.*	Case vignettes and medical records	Focussed analysis through predefined checklists to assess case vignettes and medical records. The checklist focussed on: elements of the problem analysis, use of shared-decision making during goal setting, intervention delivery (consistency between treatment plan and intervention goals, consistency between goals and intervention delivery, and interdisciplinary cooperation)
*Fidelity*: *Quality of intervention delivery.* *Adaptations*: *Changes that undermine fidelity.*	Medical records from OT and PT professionals involved in intervention delivery using checklists	Analysis of medical records, focused on: - Description of intervention delivery and adaptations - Consistence of the intervention goals with the problem analysis - Professionals’ evaluation of intervention delivery
2.Context of intervention delivery: *External elements influencing implementation or effects.*	Focus groups and interviews with professionals involved in intervention delivery, using a structured topic list	Content analysis on elements influencing implementation or effects Focus group evaluation of the Social Fitness Programme, topics: - The guideline - Individual knowledge, skills and behaviour - Client and caregiver factors - Interactions with other professionals - Incentives and recourses - Required organisational changes
3.Impact of intervention delivery: Potential *impact of intervention delivery* on participants	Interviews with client-caregiver couples assigned to the intervention group, using a structured topic list	Content analysis on participants’ evaluations on participating in the study. Interview topic list: - How did you evaluate participation in the Social Fitness Programme? - Did you gain anything? - Were your expectations met? - What should be changed to improve the programme?

*OT Occupational Therapy, PT Physiotherapy*

2. To gain understanding in the *context of intervention delivery*, all healthcare and welfare professionals involved in intervention delivery were interviewed: they participated in a focus group and those unable to join were interviewed face-to-face. The focus groups were structured using a topic guide and conducted by the researcher (HD; trained as moderator) and observed by the research assistant (DV).

3. To get insight in the *impact of intervention delivery* from clients and caregivers who were assigned to the RCTs’ intervention group and who completed all measurements were interviewed. These structured interviews were conducted by the research assistant (DV; trained as interviewer) at the clients’ or caregivers’ home.

### Data analysis

We performed quantitative and qualitative drop-out analysis for our intervention (Table 1). Regarding *implementation*, a content analysis was performed on telephone interview records to get information on recruitment. The focused analysis of adherence, fidelity and adaptations was performed on the case vignettes and medical records by the two researchers (HD,DV) independently. The answers on the open-ended questions of the case vignettes were scored on a predefined list of possible answers, creating a total percentage of intervention delivery. The OT and PT medical records were scored using a predefined list with quality criteria for the SF Programme. Scores were discussed until consensus was reached.

Focus groups and interviews on the *context of intervention delivery* and on *the impact of intervention delivery* were recorded, transcribed and analysed by two researchers (HD;DV) using Atlas.ti 7.1.4. The transcripts were thematically analysed through a content analysis [38]. The main researcher (HD) coded the focus groups and interview transcripts, and coding was checked by the research assistant (DV). The initial coding results were reviewed, discussed, and refined until consensus was reached on all codes. This resulted in identification of main themes and categories, which were discussed in project team meetings with all authors. Consecutively, we applied a checklist [39] to map professionals’ experiences and opinions regarding the *context of intervention delivery*. Qualitative data on participants’ *impact of intervention delivery* was compared to their intervention goals and medical records to get insight in the reasons for not finding effects on our primary outcome measure.

## Results

### 1) implementation

#### Reach and recruitment

Within a time frame of fifteen months 60 client/caregiver couples were informed on the study; 11 of them did not meet inclusion criteria and study participation was declined by 32 couples, which is a decline rate of 65,3% (qualitative drop-out analysis). In all, seventeen couples were included in the intervention and they were randomly assigned to the intervention group (*n* = 8) or the control group (*n* = 9).

Analysis revealed the recruitment difficulties originate from two main causes (quantitative drop-out analysis). First, a lack of internal motivation to increase social participation expressed by people with cognitive problems. Second, caregivers were often overburdened and referring professionals feared that this burden would increase if they would participate in this study. Other reasons for study decline mentioned were acute physical problems which required frequent hospital visits and denial of cognitive problems by the client.

#### Adherence

Adherence to intervention guidelines was on average sufficient for Occupational Therapists (OTs) both before and after intervention delivery (Table 2). Physiotherapists (PTs) scored insufficient before intervention delivery, however average scores after intervention delivery were sufficient.

**Table 2:** Adherence to intervention guidelines

	Before intervention delivery: Range% (average%)	After intervention delivery: Range% (average%)
Occupational Therapy (OT)	4 OTs: 61–75 (70)	8 OT records: 48–86 (69)
Physiotherapy (PT)	3 PTs: 35–58 (46)	3 PT records: 58–82 (68)

#### Fidelity and adaptations

All couples from the intervention group (*n* = 8) completed OT training within six months of intervention delivery (Table 3). Only three clients (38%) received PT training within the SF Programme, while based on frailty and mobility scores PT was indicated for all eight clients. One client was referred to PT but declined participation, and four clients continued their own regular PT treatment independent from the SF programme. Four couples already received guidance from a Dementia Casemanager before the start of the intervention, and one couple was assigned to a one during the intervention. Therefore, the coordinating OT discussed these cases with Welfare Professionals and decided together to involve the Dementia Casemanager instead of the welfare professional.

**Table 3:** Fidelity of intervention delivery

	Participants receiving intervention elements/ total number of participants in the intervention group
Received Occupational Therapy intervention (COTiD)	8/8
Received Physiotherapy intervention (Coach2Move)	3/8
Received welfare intervention	3/8

### 2) context of intervention delivery

All professionals involved in intervention delivery participated in one of two organised focus groups, in Nijmegen (*n* = 8 participants) or Deventer (*n* = 5 participants), or they were interviewed face-to-face (*n* = 3). In total, five OTs, three PTs, four Dementia Casemanagers, three Welfare Professionals and one Practice Nurse participated. Analysis of the data revealed barriers and facilitators influencing implementation and effects of the Social Fitness programme (Table 4). Emerging themes and categories were in agreement with the checklist designed by Flottorp and colleagues [39], which is a comprehensive integrated checklist of determinants of practise (TICD-checklist).

**Table 4:** Professionals’ experiences with intervention delivery

	Barriers for intervention delivery^*a)*^	Facilitators for intervention delivery^*a)*^
Theme 1	Social Fitness Programme guideline factors	
Categories theme 1	- Intervention length too short for structural behaviour change	- Professionals were motivated to participate in the SF study - *Goal setting focused the intervention* - *Additional attention for caregiver*
Theme 2	Individual health professional factors	
Categories theme 2	- Lack of clarity regarding own role during intervention delivery - Reservations in referring clients to welfare professionals - Little experience in SF programme performance - *Illness of volunteer*	- Professionals put more effort into treatment as a result of their clients participating in research - *GP supported participation in the SF programme and coordinated all care initiatives*
Theme 3	Client and caregiver factors	
Categories theme 3	- Lack of internal motivation to increase social participation expressed by people with cognitive problems - Increased caregiver burden during intervention delivery - Changing needs (i.e. as a result of physical problems) - Client was unwilling to transfer from own PT to intervention PT - *Caregiver had limited time available* - *Caregiver/client had difficulties in handling cognitive decline* - *Client had difficulties in prioritising*	- Expressed need for support to maintain or increase functioning in the home environment - Clients’ motivation to contribute in research and therefore participate in the SF programme (i.e. contribute to society) - *Client was motivated to participate (*i.e. *to prevent necessity of client for going to day-care; happy to share her story)*
Theme 4	Professional interactions	
Categories theme 4	- Suboptimal sharing of information among SF professionals - *Lack of coordination, too many people involved alongside SF Programme* - *Difficulties in reaching WP*	- Collaboration improved during the study - *Professionals were already used to working together* - *Dementia Casemanager motivated clients for OT*
Theme 5	Incentives and resources	
Categories theme 5	- Limited availability of organised social activities in the community which suit the participants with cognitive problems - *Lack of PT reimbursement by health insurance*	- Not applicable
Theme 6	Organisational resources	
Categories theme 6	- Rearrangement resulted in discontinuity of welfare professionals	- Not applicable

^*a*^ *Italic barriers and facilitators originate from OT and PT medical records*

*SF Social fitness, GP General Practitioner, OT Occupational Therapy, PT Physiotherapy*

Most barriers were related to client and caregiver factors: lack of clients’ motivation to increase social participation and to transfer to an intervention PT, increased caregiver burden, changing needs and decrease of capacity causing a focus shift and adding intervention goals during intervention delivery. Also, because of limited inclusion, professionals had little experience in intervention delivery and they were unable to get a routine. Moreover, working together in a multidisciplinary team with several different professionals was challenging. In most cases, the involved PTs where other people than the PTs who were trained in the SF protocol and involved in our study, because clients were unwilling to switch to a different PT. Additionally, the Dementia Casemanagers and the Practise Nurse were not trained in the SF protocol, although they were involved in guiding SF programme participants instead of the Welfare professionals. Most important facilitators for intervention delivery were related to client and caregivers’ motivation to accept support for enabling to function in their own home environment, and their motivation to contribute to research by participating in the study. Also, involved professionals who were highly motivated to participate in the study, were facilitators for intervention delivery.

### 3) impact of intervention delivery

To get insight in the impact of intervention delivery, we interviewed the participants who were allocated to the intervention group and who completed t2 measurements: one caregiver (i7) and four couples (i2- i3- i4- i6). All participants but one (the caregiver form couple i6) were satisfied with the results from the intervention after t2. These fairly positive evaluations with regard to intervention delivery did not result in improvements on group level in terms of results on the primary outcome which was used in this study (results are shown in Additional file 1). To get insight in the reasons for this incongruence, we performed additional qualitative analysis on the interview data as part of this process evaluation.

For all participants except one caregiver (i4), quantitative analyses revealed a (partially) mismatch between formulated goals and activities initiated during the SF Programme. This mismatch was for four clients (i2, i3, i4, i7) related to the deterioration of their cognitive and/or physical problems, which led to a shift in intervention goals and adaptation of the intervention plan. For two participants (caregiver i3, client i6) it was associated with difficulties to formulate and evaluate own personal goals. For two caregivers (i2 and i7), not all personal goals formulated at baseline were given attention during the SF Programme. Table 5 illustrates the mismatch between goal setting and intervention delivery for two clients who participated in the Social Fitness study, which contributed to the fact we did not find effects on group level.

**Table 5:** Examples of the mismatch between goal setting and intervention delivery

- Client i2 described positive experiences with participating in a fall-prevention training as part of the SF Programme, however this was not reflected in the primary outcome score as they did not change. Analysing the personal goals revealed that these did not target decreasing fall accidents but they focused instead on the clients’ wish of being in charge and making own decisions, riding a bike and travelling. This revealed a mismatch between goal setting and intervention delivery. - Client i3 described participation in new social activities, but this was not reflected in primary outcome scores. The increase in participation concerned an activity (day care) different from the activities on which goals were formulated (walking, riding a bike, playing a pool game, travelling). The formulated goals were too difficult to attain due to deterioration of the clients’ situation. This revealed a mismatch between goal setting and intervention delivery; the formulated goals were not realistic and therefore goals were changed during intervention delivery.	

## Discussion

Although the Social Fitness Programme was developed using scientific evidence, expert opinions and stakeholder needs (involving healthcare and welfare professionals, and caregivers of people with cognitive problems), and although results on feasibility seemed promising [9], we were unable to overcome implementation barriers: over 65% of the people who were referred to the effectiveness study declined participation As a result, our intervention reach was minimal and we felt the necessity to stop the inclusion and to analyse the process and barriers thoroughly. Our analysis during the process evaluation revealed the high decline rate during recruitment was mainly caused by a lack of internal motivation to increase social participation expressed by people with cognitive problems. From our previous studies [9, 25], we knew this mechanism played a role. However, we were unaware of the scale of this problem and we expected that caregivers, as they were often dissatisfied with the decreased social participation of the people they cared for, would be willing to participate and would persuade the person they cared for to participate in the study.

While recruitment difficulties are often seen in research [29–33], and interventions on social participation more often only reach only a small minority of the targeted population [40], the decline rate of our study was very high. While the single OT [10, 11, 37] and PT interventions [12–14], which were incorporated in the Social Fitness programme dealt with implementation difficulties as well, sufficient participants could be included and effectiveness of these interventions was established. Besides the single interventions being less complex, another possible reason for their success was the focus on activities of daily living, taking into account the relevance for social interaction, which is experienced as less threatening by participants with cognitive problems. We therefore hypothesize the way we framed the intervention during recruitment (social participation improvement) was suboptimal and too direct, threatening peoples’ autonomy because they are ‘accused’ of being unable to self-manage and seem to be in need for help [41]. The feeling of being unable to adequately contribute to social interactions seemed to be causing embarrassment. Framing the intervention on managing abilities in daily life in the context of decline would probably have appealed to people more. Activities of daily living such as getting dressed and preparing breakfast act as a precondition to the performance of social participation [4, 25], and therefore the focus of intervention delivery can remain unchanged. However, based on recent evidence we do suggest to incorporate behavioural coping strategies, as this is recently shown to be effective in social participation promotion [42].

Our process evaluation also revealed that besides minimal reach and recruitment difficulties, we faced more problems with regard to implementation which made it inadequate: although adherence Other implementation difficulties were related to barriers on all known healthcare levels, which was confirmed by participating professionals. A major problem in measuring effects was the shift from initial intervention goals during intervention delivery, as our primary measurement focussed on the recurrent measurement of the initial set goals.. Also, in many cases, other professionals (PTs and Dementia Casemanagers) who were not involved in the study and therefore not trained in the SF protocol, were involved in intervention delivery, which acted as a barrier for good intervention delivery. Implementation was insufficiently incorporated in existing networks.

The lack of participants to meet power calculations inhibited the effectiveness evaluation of this complex intervention. This process evaluation revealed the lack of effect was the result of a major implementation failure, rather than genuine ineffectiveness [28]. To our knowledge, no other studies aiming at social participation improvement of people with cognitive problems and their caregivers through a person-centred, individualised and community-based intervention exist.Only few effective social participation interventions really reach vulnerable populations and are implemented in practice [43]. These effective interventions were not directed at people with dementia, and resulted more in facilitating of daily activities than older adults’empowerment or community integration. Also, effects on social participation were often not considered. For example, a person-centred activity-focused case management intervention study directed at frail older adults, did not establish effectiveness recently [44]. This study showed that an intervention directed at promoting physical activity does not automatically increase social participation. More research on person-centred and community-based interventions to improve social participation in elderly people with dementia and their caregivers is therefore recommended Our intervention, the Social Fitness study, incorporates only elements known to be effective in psychosocial interventions in dementia care. We therefore believe in the potential effectiveness of our programme, but we do have to find solutions to overcome the implementation barriers we were faced with. This study adds new knowledge to this field of research, which should be used in further research to prevent and overcome these implementation barriers.

In all, the tailor-made Social Fitness Programme did not fit in three ways. *First*, offering an intervention explicitly focused at improving social participation did not fit with clients and caregivers. Managing and coping with the inevitable decline on daily basis could be a better starting point for intervention, instead of directly focussing on active social participation. *Second*, the intervention seems to be too complex to implement in the way it was designed, and as a result implementation was inadequate. This is a result of involvement and interactions between three different professionals at one hand, and changing needs, increased decline and interactions between clients and their caregivers at the other hand. Difficulties arose especially when goals on social participation which were set at the start of the intervention appeared to be too difficult to attain. We therefore suggest to incorporate one leading professional who analyses the situation on all domains, including social participation, and sets priorities, and who then involves other professionals no sooner than possible: a step-by-step approach, for goal setting and intervention delivery. *Third*, there is a tension between the offering of a tailor-made intervention and evaluating it through a fixed study design. As a result, the follow-up measurements evaluated merely unfinished treatments and overall outcomes at fixed times. A participatory design would have fitted the effectiveness evaluation of this intervention better [45]. Participatory Action Research focuses on social processes and collaboration with participants to get insight in actual changes in practise [46].

## Conclusions

The Social Fitness study did not fit in three ways. First, framing the intervention on social participation promotion was as threatening to clients. The feeling of being unable to adequately contribute to social interactions seemed to be causing embarrassment. Second, the intervention seemed to be too complex to implement in the way it was designed. Third, there is a tension between the offering of a personalised tailor-made intervention and evaluation through a fixed study design.

## Additional file

Additional file 1: Original study design and outcomes of the Social Fitness Programme RCT**.** We designed an effectiveness study (RCT) to evaluate the Social Fitness Programme. As a result of the high amount of study decline in relation to the participants who gave informed consent, study inclusion was terminated. As a result of limited inclusion it was not feasible to perform the linear mixed models for the primary outcomes as planned. We therefore performed explorative descriptive analyses on our data. Additional file 1 contains the study design of the RCT and the descriptive results of our primary and secondary outcomes. (DOCX 74 kb)

## Abbreviations

COPM: Canadian Occupational Performance Measure
COTiD: Community Occupational Therapy in Dementia
GP: General Practitioner
IQCODE-N: Informant Questionnaire on COgnitive Decline in the Elderly
MMSE: Mini-Mental State Examination
MRC: Medical Research Council
OT: Occupational Therapist
PT: Physiotherapist
RCT: Randomised Controlled Trial
SF Programme: Social Fitness Programme
