# Supplementary material for: Evaluating the social fitness Programme for older people with cognitive problems and their caregivers: lessons learned from a failed trial
Source: BMC Geriatr. 2018 Oct 4;18:237. doi: 10.1186/s12877-018-0927-8 (PMC6172728; doi:10.1186/s12877-018-0927-8)
Supplement: Supplementary file 1 — Original study design and outcomes of the Social Fitness Programme RCT. We designed an effectiveness study (RCT) to evaluate the Social Fitness Programme. As a result of the high amount of study decline in relation to the participants who gave informed consent, study inclusion was terminated. As a result of limited inclusion it was not feasible to perform the linear mixed models for the primary outcomes as planned. We therefore performed explorative descriptive analyses on our data. Additional file 1 contains the study design of the RCT and the descriptive results of our primary and secondary outcomes. (DOCX 74 kb) [file 12877_2018_927_MOESM1_ESM.docx]

**Additional file 1**

**Original study design and outcomes of the Social Fitness Programme RCT**

***Study design***The design of the effectiveness study was a single blind RCT with measurements at baseline, 3 months and 6 months after the start of the intervention. Individual client-caregivers were randomised at a 1:1 ratio, using block randomisation with block size 4, to the intervention or control group. They were stratified for the practice where the intervention was offered. In the intervention group, clients and their caregivers participated in the SF Programme; those assigned to the control group received usual care by their general practitioner and were placed on a waiting list to participate in the intervention following their last assessment (after 6 months). The SF Programme was considered to be a success when the intervention group was superior to the control group in (at least) one of two outcome measures, and at least not inferior in the other one. The sample size calculation for this success criterion is elaborated on elsewhere [1].

***Participants and randomisation***We aimed to included 92 Clients and caregivers couples who experienced a reduction in social participation. Recruitment took place in two regions (Nijmegen and Deventer) in The Netherlands. *Clients* were eligible if they lived at home, wished to improve their social participation and suffered from cognitive problems defined as: dementia diagnosis (MMSE ≥10; [2] or memory problems signalled by the referring professional (Mini-Mental State Examination (MMSE) 10-24) or with a primary caregivers’ score of ≥3.6 on the Questionnaire on COgnitive Decline in the Elderly (IQCODE-N) [3] (only for clients with high intelligence or high levels of education resulting in an MMSE-score between 25 and 30). Moreover, also *clients’ primary caregivers* who wished to maintain or improve their own social participation or the social participation of the people they cared for were eligible. Participants were excluded if they: were unable to formulate goals for social participation during the screening interview, were not capable of completing the self-assessment forms (i.e. due to language problems); suffered from co-morbidity with symptoms that interfered with actively taking part in the intervention (e.g. behavioural and psychological symptoms of dementia (BPSD), severe heart condition); used less than three months the same dose of medication which influenced their cognition (cholinesterase inhibitor or memantine); were in a palliative phase of illness; had acute illness with hospital indication; received already physiotherapy according to the Coach2Move protocol or occupational therapy according to the COTiD-programme in the last 6 months, or participated in other health research.

Potential eligible participants were informed on the study by healthcare and welfare professionals using verbal and/or written (leaflet) information on the study. When willing to participate, potential eligibility was determined by the research assistant by phone. In case of potential eligibility, an appointment for a home visit was made with the client and caregiver, in which they were provided with information on research participation and study procedures (verbal and in writing). When still willing to participate, a second appointment was made at minimum one week later, in which the research assistant checked further inclusion criteria (i.e. assess cognition if no valid MMSE-score was available) and obtained signed informed consent forms from both the client and the caregiver.

Randomisation took place after the baseline measurements administered during the second home visit and was carried out by a statistician not involved in the study (ST), using a computerised randomisation protocol. The main researcher not involved in data collection (HD) informed clients and their caregivers about their allocation by means of a concealed envelope, and informed the occupational therapist and physiotherapist, and the general practitioner of the person with cognitive problems about group allocation. When allocated to the intervention group, the OT arranged a first appointment with the client and caregiver immediately, when they were allocated to the control group the first appointment with the OT took place after six months. Follow-up measurements were performed by a research assistant not aware of group allocation (DV), and participants were asked by the main researcher (HD) not to reveal information on their allocation.

***Measurements***RCT data on observational and self-report outcome measures were collected during home visits, using interviews with the client (face-to-face) and separately with the caregiver (face-to-face) and through written questionnaires filled in by the caregiver. Assessments were performed by a blinded research assistant who was not aware of group allocation at baseline (t0), after three months (t1), and after six months (t2) follow-up. Table 1 shows an overview of the outcome measures for both the client and the caregiver.

***Data analysis***We planned to analyse mean outcomes at baseline, t0 and t1 using descriptive statistics. Differences between baseline and three months were to be computed by a linear mixed model for repeated measurements with age, sex and time (t0, t1,t2) as covariates. The Canadian Occupational Performance Measure (COPM) performance and COPM satisfaction scores were planned to be tested separately for clients and caregivers. In case of significant improvements at three months, we planned to carry out secondary conditional analyses on this primary outcome measure at six months. The analyses followed the principle of intention to treat for all participants who at least had one session of OT (intervention group). Dropout would be primarily dealt with by the linear mixed model, which assumed missing data is missing at random given the covariates. We planned to perform an economic evaluation including calculating the differences in total costs in the control and intervention group at three and six months using the Resource Utilization in Dementia (RUD) [4] and full-cost prices [5]. We also planned to calculate cost effectiveness using the EuroQuol (EQ-5D) [6] at three and six months, and costs effectiveness for successful treatment outcome at three months.

| **Outcome** | **Instrument** | **Assessment** |
| --- | --- | --- |
| **Client with cognitive problems** | | |
| *Primary outcome* | | |
| Self perceived performance and satisfaction on social participation goals | COPM [7]  higher mean score = improved perceived performance/ more satisfaction with social participation | Face-to-face |
| *Secondary outcomes* | | |
| Mobility | TUG [8]  higher score = decreased mobility | Face-to-face |
| Quality of life | DQoL [9]  higher score = better qual. of life | Face-to-face |
| Health related quality of life | EQ-5D [6]  higher score = better health related quality of life | Face-to-face |
| Resource utilization | RUD [4]  higher score = more resource utilization | Questionnaire by caregiver |
| *Covariates* | | |
| Frailty | EFIP [10]  higher score = more frailty | Face-to-face |
| **Caregiver** | | |
| *Primary outcome* | | |
| Self perceived social participation | COPM performance/satisfaction | Face-to-face |
| *Secondary outcomes* | | |
| Caregiver Burden | SCQ [11]  higher score = better sense of competence | Questionnaire |
| Quality of life | DQoL | Questionnaire |
| Health related quality of life | EQ-5D | Questionnaire |
| Resource utilization | RUD  higher score = more resource utilization | Questionnaire |
| *Covariates* | | |
| Socio-demographics | Questionnaire | Questionnaire |

**Table 1** Primary and secondary outcomes, measurement instruments and assessment

**Results**

Between January 2014 and March 2015, sixty client/caregiver couples were informed about the study and assessed for eligibility (Figure 1). Eleven couples were excluded because they did not meet inclusion criteria. We aimed to include 92 couples for full RCT; however after an inclusion period of 15 months it appeared that of 49 participants fulfilling inclusion criteria, 32 declined participation and only 17 couples could be included. As a result of the high amount of study decline in relation to the participants who gave informed consent, study inclusion was terminated. Figure 1 shows the trial Flowchart.

**Figure 1. Trial Flowchart**

Not meeting inclusion criteria (n=11)

Declined to participate (n=32)

Control group allocated to waiting list (n=9)

Intervention group allocated to SF Programme (n=8)

Assessed for eligibility (n=60)

t0: baseline measurement
(n=17)

t1: measurement at 3 months (n=8)

Lost to follow-up (n=0)

Randomisation

(n=17)

t2: measurement at 6 months (n=7)

Lost to follow-up (n=2)

- Death (n=2)

t2: measurement at 6 months (n=6)

Lost to follow-up (n=2)

- Lost interest (n=2)

t1: measurement at 3 months (n=9)

Lost to follow-up (n=0)

The seventeen couples included were randomly assigned to the intervention group (n=8) or the control group (n=9). All couples received follow-up measurement at three months (t1 measurement). Four couples were lost to follow-up at six months (t2 measurement); two couples from the intervention group, and two couples from the control group. Demographic characteristics of participants did not significantly differ between groups and are shown in Table 2.

|  | **Intervention group (n=8)** | **Control group (n=9)** |
| --- | --- | --- |
|  | **n (%) or mean (SD)** | **n (%) or mean (SD)** |
| **Age (years)** clients  **Age (years)** caregivers | 81 (9.78)  71 (10.69) | 81 (7.47)  68 (12.7) |
| **Gender (% men)** clients  **Gender (% men)** caregivers | 5 (62.5)  2 (25) | 4 (44.4)  2 (22.2) |
| **Relation client - caregiver**  Husband – Wife  Wife - Husband  Parent – Child  Women – Sister | 5 (62.5) 1 (12.5) 2 (25.0) - | 3 (33.3)  1 (11.1)  4 (44.4)  1 (11.1) |
| **Education** clients  Lower level  Middle level  Higher education  **Education** caregivers  Lower level  Middle level  Higher education | 3 (37.5)  4 (50.0)  1 (12.5)  3 (37.5)  2 (25)  3 (37.5) | 4 (44.4)  4 (44.4)  1 (11.1)  3 (33.3)  3 (33.3)  3 (33.3) |
| **MMSE** clients (0-30) | 23.38 (20.50-27.75) | 22 (16.50-26.50) |
| **IQ-code** clients (1-5)* | 3.84 (3.77-4.22) | 4.01 (3.79-4.26) |

* Only when MMSE >24 IQ-code by proxy (*n*=8)
**Table 2** Demographic characteristics

As a result of limited inclusion it was not feasible to perform the linear mixed models for the primary outcomes (COPM performance and COPM satisfaction) as planned. Instead we performed descriptive analyses of the primary and secondary outcomes at baseline and three months follow-up (Table 3). Since data was skewed, categorical and continuous variables were described using proportions and median with quartiles (Q). Experimental and control group were largely comparable at baseline, with exception of caregivers from the control group who reported higher COPM scores at baseline compared to the intervention group, with a difference of 1.5 point. Clients from the experimental group reported slightly higher COPM performance scores (0.75 point).

Primary outcomes at t1 (Table 3) showed clinically relevant (i.e. ≥1 point difference) COPM scores for clients and caregivers in both intervention and control group, except for caregivers’ COPM scores on satisfaction which showed a non-clinically relevant decrease. Clients from intervention group reported slightly larger improvements compared to the control group. At t1, improvements were larger for clients’ COPM satisfaction scores compared to clients’ COPM performance scores. At t1, caregivers from the intervention group reported a larger improvement on COPM scores (2.25 on performance and 2.75 on satisfaction) compared to those from the control group (0.25 increased performance and 0.12 decrease in satisfaction).

Secondary outcomes showed diffuse results. Although we saw small differences in numerical scores, none of the changes were significant or clinically relevant on group level (Table 3).

| **Clients** |  | **Baseline** | **Three months** | **Caregivers** |  | **Baseline** | **Three months** |
| --- | --- | --- | --- | --- | --- | --- | --- |
|  |  | Median (Q1-Q3) | Median (Q1-Q3) |  |  | Median (Q1-Q3) | Median (Q1-Q3) |
| **COPM** (1-10)  *Performance*  *Satisfaction* | I  C  I  C | 3.75 (1.00-5.00)  3.00 (1.00-3.71)  4.75 (3.50-5.33)  4.63 (3.32-5.88) | 5.25 (3.50-6.00)  4.15 (1.50-5.88)  6.55 (4.69-8.35)  6.25 (3.81-9.25) | **COPM** (1-10)  *Performance*  *Satisfaction* | I  C  I  C | 3.50 (1.67-5.00)  5.00 (3.75-5.60)  3.50 (2.50-6.00)  5.00 (3.25-5.40) | 5.75 (2.62-7.68)  5.25 (4.63-5.94)  6.25 (5.17-9.50)  4.88 (4.37-6.50) |
| **DQoL** (1-5)  Positive affect  Negative affect  Feelings of belonging  Self-esteem  Sense of aesthetics | I  C  I  C  I  C  I  C  I  C | 3.75 (3.29-4.00)  3.67 (3.17-4.00)  2.59 (2.18-3.07)  2.18 (1.77-3.45)  3.67 (3.08-4.00)  3.67 (2.50-4.00)  3.50 (3.13-3.94)  3.25 (3.00-4.25)  4.30 (3.35-4.55)  4.00 (3.40-4.10) | 3.83 (3.21-4.13)  3.58 (3.33-3.92)  2.41 (1.86-2.77)  2.45 (1.64-2.91)  3.67 (2.75-4.00)  3.83 (3.08-4.00)  3.63 (2.81-4.38)  3.50 (3.25-4.00)  3.80 (2.80-4.00)  4.00 (2.90-4.20) | **DQoL** (1-5)  Positive affect  Negative affect  Feelings of belonging  Self-esteem  Sense of aesthetics | I  C  I  C  I  C  I  C  I  C | 3.17 (2.83-3.83)  3.50 (3.25-4.00)  2.55 (2.18-3.36)  2.77 (1.68-3.61)  4.00 (4.00-4.58)  4.00 (3.67-4.50)  4.25 (4.00-4.44)  3.75 (3.38-4.38)  3.80 (2.35-4.15)  4.40 (3.10-4.90) | 3.50 (2.29-3.79)  3.92 (3.17-4.00)  2.36 (2.36-3.00)  2.32 (2.11-2.80)  4.00 (3.50-4.58)  4.17 (3.33-4.58)  3.75 (2.88-3.75)  4.13 (3.13-5.00)  3.60 (2.80-4.00)  4.00 (4.00-4.00) |
| **TUG** (0-...) | I  C | 16.70 (14.53-21.15)  12.85 (11.80-21.58) | 18.80 (12.88-22.80)  11.35 (10.95-19.48) | **SCQ** (1-5) | I  C | 3.83 (2.50-4.00)  3.86 (2.61-4.13) | 3.94 (2.17-4.17)  3.89 (3.71-4.42) |
| **EFIP** (0-1) | I  C | 0.28 (0.26-0.30)  0.21 (0.14-0.30) | 0.35 (0.27-0.40)  0.20 (0.15-0.23) |  |  |  |  |

**Table 3** Primary and secondary outcomes at baseline (t0) and 3 months follow-up (t1). Q=Quartiles

Table 4 presents the results of our analyses using descriptive statistics on data for individual clients and caregivers. For both clients and caregivers, most clinically relevant successful outcomes were observable in their satisfaction scores at t1. However, these improvements decreased at t2 (Table 4).

|  | **Performance client** | | **Satisfaction client** | | **Performance caregiver** | | | **Satisfaction caregiver** | | |
| --- | --- | --- | --- | --- | --- | --- | --- | --- | --- | --- |
|  | **t0-t1** | **t0-t2** | **t0-t1** | **t0-t2** | **t0-t1** | | **t0-t2** | **t0-t1** | **t0-t2** | |
| **Intervention group** | | | | | | | | | | |
| i1 | 0,3 | - | 2,8 | - | -1,0 | - | | -1,5 | | - |
| i2 | -0,7 | -1.7 | 3,0 | -0.1 | 1,7 | 0,3 | | -1,0 | | -1,7 |
| i3 | 1,5 | -1,0 | -0,5 | -0,5 | - | - | | - | | - |
| i4 | 0,3 | -0,1 | 1,8 | 1,8 | 5,0 | 3,0 | | 4,0 | | 2,0 |
| i5 | 7,5 | 3,5 | 3,5 | 3,0 | -2,0 | -6,0 | | -1,0 | | -3,0 |
| i6 | - | - | - | - | 3,0 | 1,0 | | 4,0 | | 3,5 |
| i7 | 2,5 | - | - | - | 0,7 | 0,3 | | 4,0 | | 4,0 |
| i8 | 0,0 | - | -0,5 | - | 0,1 | - | | 7,0 | | - |

**Legend**

| Superiority: ≥1 point improvement |
| --- |
| Inferiority: ≥ 1 pint decline |
| No change: < 1 point improvement |

**Table 4** Mean changes at t1 and t2 for primary Canadian Occupational Performance Measurement scores (COPM-scores)

**References**

1. Donkers H, Graff M, Vernooij-Dassen M, Nijhuis-van der Sanden M, Teerenstra S: **Reducing sample size by combining superiority and non-inferiority for two primary endpoints in the Social Fitness study**. *Journal of clinical epidemiology* 2017, **81**:86-95.

2. Vertesi A, Lever JA, Molloy DW, Sanderson B, Tuttle I, Pokoradi L, Principi E: **Standardized Mini-Mental State Examination. Use and interpretation**. *Canadian family physician Medecin de famille canadien* 2001, **47**:2018-2023.

3. Ayalon L: **The IQCODE versus a single-item informant measure to discriminate between cognitively intact individuals and individuals with dementia or cognitive impairment**. *Journal of geriatric psychiatry and neurology* 2011, **24**(3):168-173.

4. Wimo A, Jonsson L, Zbrozek A: **The Resource Utilization in Dementia (RUD) instrument is valid for assessing informal care time in community-living patients with dementia**. *The journal of nutrition, health & aging* 2010, **14**(8):685-690.

5. Hakkaart-Van Roijen L, Tan S, Bouwmans C: **Handleiding voor kostenonderzoek. Methoden en standaard kostprijzen voor economische evaluaties in de gezondheidszorg. [Guideline for cost evaluation.]**. Rotterdam: Erasmus Universiteit Rotterdam: Instituut voor Medical Technology Assessment; 2010.

6. Williams A: **Euroqol - a New Facility for the Measurement of Health-Related Quality-of-Life**. *Health policy* 1990, **16**(3):199-208.

7. Law M, Baptiste S, McColl M, Opzoomer A, Polatajko H, Pollock N: **The Canadian occupational performance measure: an outcome measure for occupational therapy**. *Canadian journal of occupational therapy Revue canadienne d'ergotherapie* 1990, **57**(2):82-87.

8. Podsiadlo D, Richardson S: **The timed "Up & Go": a test of basic functional mobility for frail elderly persons**. *Journal of the American Geriatrics Society* 1991, **39**(2):142-148.

9. Brod M, Stewart AL, Sands L, Walton P: **Conceptualization and measurement of quality of life in dementia: the dementia quality of life instrument (DQoL)**. *The Gerontologist* 1999, **39**(1):25-35.

10. de Vries NM, Staal JB, Olde Rikkert MG, Nijhuis-van der Sanden MW: **Evaluative frailty index for physical activity (EFIP): a reliable and valid instrument to measure changes in level of frailty**. *Physical therapy* 2013, **93**(4):551-561.

11. Jansen AP, van Hout HP, van Marwijk HW, Nijpels G, Gundy C, Vernooij-Dassen MJ, de Vet HC, Schellevis FG, Stalman WA: **Sense of competence questionnaire among informal caregivers of older adults with dementia symptoms: a psychometric evaluation**. *Clinical practice and epidemiology in mental health : CP & EMH* 2007, **3**:11.
